# Implementation and management outcomes of pharmacogenetic CYP2C19 testing for clopidogrel therapy in clinical practice

**DOI:** 10.1007/s00228-020-03050-4

**Published:** 2020-11-26

**Authors:** Stefan Russmann, Ali Rahmany, David Niedrig, Karl-Dietrich Hatz, Katja Ludin, Andrea M. Burden, Lars Englberger, Roland Backhaus, Andreas Serra, Markus Béchir

**Affiliations:** 1 drugsafety.ch, Seestrasse 221, 8700 Küsnacht, Switzerland; 2 Institute of Internal Medicine and Nephrology, Clinic Hirslanden, Zurich, Switzerland; 3 Center for Internal Medicine, Clinic Hirslanden, Aarau, Switzerland; 4 Pharmaceutical Sciences, Swiss Federal Institute of Technology Zurich (ETHZ), Zurich, Switzerland; 5 Hospital Pharmacy, Clinic Hirslanden, Zurich, Switzerland; 6 INTLAB AG, Zurich, Switzerland; 7 Labor Risch, Mocelular Genetics, Berne, Switzerland; 8 Cardiac Surgery, Clinic Hirslanden, Aarau, Switzerland; 9 Stroke Center and Clinic for Neurology, Clinic Hirslanden, Zurich, Switzerland

**Keywords:** Pharmacogenetics, CYP2C19, Clopidogrel, Cardiology, Clinical pharmacology

## Abstract

**Purpose:**

The antiplatelet prodrug clopidogrel is bioactivated by the polymorphic enzyme CYP2C19. Prospective clinical studies demonstrated an association between CYP2C19 loss of function (LoF) variants and an increased risk of thrombotic events under clopidogrel, but pharmacogenetic (PGx) testing is not frequently implemented in clinical practice. We report our experience with PGx-guided clopidogrel therapy with particular regard to clinically relevant patient management changes.

**Methods:**

We conducted an observational study analyzing patients that underwent PGx testing for clopidogrel therapy at two Swiss hospitals. Primary outcome was the proportion of patients with clinically relevant PGx-based management recommendations and their implementation. The association of recurrent ischemic events under clopidogrel with CYP2C19 LoF variants and other factors was explored in a multivariate case-control analysis.

**Results:**

Among 56 patients undergoing PGx testing, 18 (32.1%) were classified as CYP2C19 intermediate or poor metabolizers. This resulted in 17 recommendations for a change of antiplatelet therapy, which were implemented in 12 patients (70.1%). In the remaining five patients, specific reasons for non-implementation could be identified. Recurrent ischemic events under clopidogrel were associated with LoF variants (OR 2.2, 95% CI 0.3–14.4) and several cardiovascular risk factors. Associations were not statistically significant in our small study, but plausible and in line with estimates from large prospective studies.

**Conclusion:**

PGx-guided clopidogrel therapy can identify patients with an elevated risk of ischemic events and offer evidence-based alternative treatments. Successful implementation in clinical practice requires a personalized interdisciplinary service that evaluates indications and additional risk factors, provides specific recommendations, and proactively follows their implementation.

## Introduction

Clopidogrel is a P2Y12 antagonist antiplatelet drug indicated for the prevention of cardiac, peripheral, or cerebral ischemic events, particularly after vascular interventions and in combination with low-dose aspirin as dual antiplatelet therapy (DAPT) [1–3]. In contrast to aspirin and other drugs used for DAPT such as ticagrelor or prasugrel, clopidogrel is an inactive prodrug, and its hepatic bioactivation is dependent on the polymorphic cytochrome P450 enzyme 2C19 (CYP2C19), for which there are variants with decreased (*2, *8) or increased (*17) activity [4]. Several original studies reported an association of CYP2C19 loss of function (LoF) variants with thrombotic events under clopidogrel therapy that are addressed in expert guidelines and regulatory boxed warnings [5–15]. In contrast, the CYP2C19 *17 variant with increased activity leads to greater platelet inhibition in vitro and an increased bleeding risk, whereas it does not appear to ensure greater protection against ischemic events [16]. Moreover, ticagrelor and prasugrel have also been associated with a higher risk of bleeding compared to clopidogrel [3]. While optimal DAPT is a field of ongoing research and debates [17], to date, clopidogrel remains the most commonly prescribed P2Y12 inhibitor [3], and two recent studies reported that pharmacogenetic (PGx)-guided clopidogrel therapy may achieve similar efficacy as ticagrelor and prasugrel without the cost of an increased bleeding risk [5, 14].

However, the implementation of PGx-guided clopidogrel therapy in clinical practice remains a complex challenge [18]. Our specialized clinical pharmacology services therefore established a comprehensive PGx-guided pharmacotherapy program at two Swiss hospitals. As part of this program, we not only offer CYP2C19 genotyping, but we also consult with patients and treating physicians regarding personalized management of antiplatelet therapy.

The current study describes our experience with PGx-based genotyping for clopidogrel therapy in routine clinical practice. In particular, we aimed to analyze the frequency of PGx test results with clinically relevant findings that resulted in subsequent management recommendations and changes in pharmacotherapy. Furthermore, we hypothesized that CYP2C19 loss of function (LoF) variants have a higher prevalence in patients with a previous ischemic event during clopidogrel therapy.

## Methods

### Study design

We conducted a descriptive cohort study and a nested case-control study. The primary outcome of the cohort study was the proportion of patients where pharmacogenetic testing for CYP2C19 variants had clinically relevant management implications for current or planned clopidogrel therapy in a clinical practice setting. The case-control study was nested within the population of the descriptive cohort study, and the primary outcome was the association of CYP2C19 LoF variants with recurrent thrombotic events during clopidogrel therapy. The case-control analysis was limited to patients without preemptive testing, because the finding of a CYP2C19 variant would have prevented follow-up during clopidogrel therapy.

The study protocol was reviewed and approved by the local ethics board (EKNZ project ID 2020-00565), and all included patients had signed informed consents for pharmacogenetic testing and scientific use of their health data.

### Study population, PGx patient management, and review of medical records

We included all patients who underwent PGx testing with the specific indication of clopidogrel therapy between June 2018 and May 2020 through drugsafety.ch clinical pharmacology services at the Hirslanden Hospitals in Aarau or Zurich, Switzerland. Patients were referred by treating physicians from the specialties of internal medicine, cardiology, cardiac surgery, and neurology. The indication for PGx testing was evaluated by a senior clinical pharmacologist (SR), including a personal patient consultation and a review of all medical diagnoses and current pharmacotherapy.

The referral process and clinical pharmacology consultation before PGx testing led to a selected study population with an evidence-based indication for PGx testing and typically several risk factors for recurrent thrombotic events during clopidogrel therapy. After receipt of PGx testing results including automated interpretations from the SONOGEN XP expert system (www.sonogen.eu), the clinical pharmacologist sent a written report to patients and referring physicians that included personalized PGx-based management recommendations. As part of the current study, we retrieved information on the implementation of these recommendations by contacting patients and treating physicians as necessary.

For the nested case-control analysis, cases were defined as patients with an idiopathic recurrent cardiac or peripheral thrombotic event during clopidogrel therapy. These events also constituted the indication for PGx testing. Non-idiopathic events, i.e., thrombotic events with an identifiable cause, were excluded. Controls were defined as patients without a documentation of thrombotic events during clopidogrel therapy.

For validation purposes, a senior clinical pharmacologist (SR), a senior pharmacist (DN), and a pharmacist in training (AR) reviewed all available patients’ original medical records, referral letters, pharmacotherapy prescriptions, and laboratory results.

### Genetic analysis and pharmacogenetic expert system

For PGx analyses, venous blood samples were obtained using EDTA containing Vacutainers. DNA extraction and PGx analyses were performed by Labor Risch molecular genetics laboratory, Bern-Liebefeld, Switzerland. DNA was extracted using the QIAsymphony DSP DNA Mini Kit according to the manufacturer’s instructions. The SNPs of CYP2C19 of the isolated DNA were subsequently amplified by means of the iPLEX assay which consists of multiplex-PCR, SAP reaction, and iPLEX primer extension. The modified products were then separated using the MassARRAY MALDI-TOF System by Agena Bioscience. The analysis included the following CYP2C19 associated SNPs: *rs12248560* (*17), *rs28399504* (*4), *rs41291556* (*8), *rs4244285* (*2), *rs4986893* (*3), *rs56337013* (*5), *rs72552267* (*6), and *rs72558186* (*7).

Analyzed genotypes were subsequently forwarded to SONOGEN and further processed by its XP expert system. SONOGEN XP provides an automated report with classification of the genotype into a corresponding metabolizer phenotype based on established star allele nomenclature, as well as predictions of interactions between the identified gene variants and current or potential future pharmacotherapy.

### Data analysis

Data analysis of the cohort study was descriptive with presentation of results in tables and text as appropriate. Data presented in Tables 1 and 3 refer to the time of PGx testing. For the case-control analysis, we calculated associations between recurrent thrombotic events and the presence of LoF variants as crude univariate and adjusted multivariate odds ratios along with their 95% confidence intervals (CI). For the multivariate analysis, we performed a logistic regression analysis with cases as the dependent variable and the following independent variables: LoF variants, gender, aspirin therapy in addition to clopidogrel (DAPT), history of peripheral artery disease, cerebrovascular disease, diabetes (all dichotomous variables), and age (continuous variable). Data management and analyses were performed using STATA MP version 15.1 (STATA Corporation, College Station, TX, USA).

**Table 1:** Characteristics of the study population

	All patients	CYP2C19 NM or RM	CYP2C19 IM or PM
*n* (%)	56 (100)	38 (67.9*)	18 (32.1*)
Age, *n* (%)			
< 60	6 (10.7)	4 (10.5)	2 (11.1)
60–70	14 (25.0)	11 (29.0)	3 (16.7)
71–80	20 (35.7)	14 (36.8)	6 (33.3)
> 80	16 (28.6)	9 (23.7)	7 (38.9)
Sex, *n* (%)			
Male	40 (71%)	27 (71.1)	13 (72.2)
Female	16 (29%)	11 (28.9)	5 (27.8)
eGFR** < 60 ml/min/1.73 m^2^	14 (26.4)	10 (27.8)	4 (23.5)
Indication for clopidogrel, *n* (%)			
Coronary artery disease	20 (35.7)	15 (39.5)	5 (27.8)
Peripheral artery disease	17 (30.4)	10 (26.3)	7 (38.9)
Cerebrovascular disease	18 (32.1)	13 (34.2)	5 (27.8)
Left atrial appendage closure	1 (1.8)	0 (0)	1 (5.6)
Pharmacotherapy, *n* (%)			
Aspirin	32 (57.1)	23 (60.5)	9 (50.0)
Clopidogrel	44 (78.6)	29 (76.3)	15 (83.3)
Prasugrel or ticagrelor	1 (1.8)	1 (2.6)	0 (0)
Coumarines or NOAC	17 (30.4)	8 (21.0)	9 (50.0)
Beta blockers	31 (56.4)	24 (64.9)	7 (38.9)
ACE or AT2inh	37 (67.3)	25 (67.6)	12 (66.7)
Diuretics	22 (40.0)	17 (46.0)	5 (27.8)
Proton pump inhibitors	30 (54.6)	19 (51.4)	11 (61.1)
Cholesterol lowering drugs	38 (69.1)	26 (70.3)	12 (66.7)
Coronary vasodilators	3 (5.4)	3 (8.1)	0 (0)
NSAIDs	3 (5.4)	2 (5.3)	1 (5.6)
Antidepressants, antipsychotics, or benzodiazepines	14 (25.5)	12 (32.4)	2 (11.1)
CYP2C19 inhibitor	5 (8.9)	3 (7.9)	2 (11.1)

*NM* normal metabolizer, *RM* rapid metabolizer, *IM* intermediate metabolizer, *PM* poor metabolizer

*% refers to row percentage. All other % refer to strata of column categories

**Glomerular filtration rate estimated according to CKD-EPI equation

## Results

Our study population included 56 patients with clopidogrel therapy as the primary indication for PGx testing. Patient characteristics are presented in Table 1, including a stratification over the CYP2C19 genotype, showing overall similar characteristics in normal or rapid vs. intermediate or poor metabolizers. Median age was 74 years (range 47 to 92 years), and there were more male than female patients (71 vs. 29%, respectively). Indication for clopidogrel therapy was approximately evenly distributed between coronary artery, peripheral artery, and cerebrovascular diseases, each accounting for about one third of the population. It is worth noting that the majority of patients (78.6%) were already on clopidogrel therapy at the time of referral for PGx testing of CYP2C19 variants. Thus, we were only able to perform generally more preferable preemptive testing before the start of clopidogrel therapy in the remaining 21.4% of patients. Furthermore, it is of interest that about one third of the patients also received an oral anticoagulant, which is relevant when benefits vs. risks of clopidogrel therapy are evaluated for individual patients with different CYP2C19 variants. The median number of prescribed drugs was 8 (range 2–19), and because we routinely conduct a check for interacting drugs in all our patients, we also identified 5 patients (8.9%) with a concomitant prescription of a CYP2C19 inhibitor (e.g., esomeprazole), which resulted in additional recommendations for change of the comedication.

Results of CYP2C19 PGx testing are presented in Table 2. Eighteen patients (32.1%) were carriers of alleles with LoF variants. Among those 18 patients with presumably impaired bioactivation and therefore limited efficacy of clopidogrel, we recommended a change of antiplatelet medication in 17. We did not recommend a change in one intermediate metabolizer due to an elevated bleeding risk related to inflammatory bowel disease after individual discussion with the treating physician. In the remaining 17 patients with coronary or peripheral artery disease, the recommendation was usually a switch to prasugrel, with dose reduction to 5 mg per day in patients ≥ 75 years. In some patients, e.g., with a history of stroke or transient ischemic attack (TIA) where prasugrel is formally contraindicated, our alternative recommendation could have been low-dose rivaroxaban plus aspirin depending on the indication, comorbidities, and after consultation with the treating cardiologist or neurologist [19–21].

**Table 2:** Frequencies of CYP2C19 genotypes, recommendations based on genotype, and implementation of recommendations

Phenotype/genotype	*n* (%)	PGx-based recommendation, *n* (%)	Implementation of recommendation, *n* (%)*
All patients	56 (100)	17 (30.4)	12 (70.1)
No LoF carriers		0	0
Normal metabolizer (NM)	34 (60.7%)		
*1/*1	20 (35.7%)		
*1/*17	14 (25.0%)		
Rapid metabolizer (RM)	4 (7.1%)		
*17/*17	4 (7.1)		
LoF carriers	18 (32.1)	17	12
Intermediate metabolizer (IM)	17 (30.4%)	16	9
*1/*2	12 (21.4%)		
*1/*8	1 (1.8%)		
*2/*17	4 (7.1%)		
Poor metabolizer (PM)	1 (1.8%)	1	1
*2/*2	1 (1.8%)		

*Proportion of patients where recommendation was implemented among those where a recommendation was made based on CYP2C19 genotype

Recommendations in the 17 patients with LoF variants were subsequently implemented in 12 patients. In the other 5 patients, reasons for non-implementation or adjustment of our initial recommendation included a limited remaining duration of DAPT indication after coronary intervention, concomitant full oral anticoagulation with phenprocoumon or rivaroxaban, or other risk factors for bleeding complications.

Results of the case-control analysis are presented in Table 3. Ten patients were referred to us because of a recurrent thrombotic event in spite of clopidogrel therapy. One was excluded from the case-control analysis because of a non-idiopathic peripheral thrombotic event, i.e., a lower limb thrombosis below a previously inserted peripheral stent that occurred shortly after kneeling while gardening. The CYP2C19 genotypes of the remaining 9 cases were compared in univariate and multivariate analyses to those of 34 controls without thrombotic events during clopidogrel therapy. Although statistical power was limited in our small study, the univariate (OR 1.9, 95% CI 0.4–8.7) as well as the multivariate (OR 2.2, 95% CI 0.3–14.4) analysis identified a similar point estimate for the odds ratio indicating an association of CYP2C19 LoF variants with recurrent thrombotic events. The multivariate analysis additionally calculated increased odds of recurrent thrombotic events among patients with diabetes and peripheral artery disease, whereas female gender and dual antiplatelet therapy with clopidogrel plus aspirin appeared to be protective.

**Table 3:** Association of recurrent thrombotic events under clopidogrel therapy with CYP2C19 genotype and other factors

	OR*	(95% CI)	*p*
Univariate analysis			
CYP2C19 IM or PM	1.9	(0.4–8.7)	0.40
Multivariate analysis			
CYP2C19 IM or PM	2.2	(0.3–14.4)	0.41
Age	0.9	(0.1–1.0)	0.08
Female gender	0.5	(0.05–5.87)	0.48
Diabetes	3.3	(0.4–25.2)	0.26
Peripheral artery disease	3.6	(0.4–34.1)	0.26
Cerebrovascular disease	1.3	(0.2–9.5)	0.78
Dual platelet inhibition (aspirin in addition to clopidogrel)	0.6	(0.1–4.6)	0.64

*Odds ratio for recurrent thrombotic event under clopidogrel therapy from univariate analysis and multivariate logistic regression analysis; in the multivariate analysis, age is modeled as a continuous variable, and all other variables are binary

## Discussion

Our study identified CYP2C19 LoF variants in 32.1% of our study population with current or planned clopidogrel therapy, and in the subpopulation of our case-control analysis, the point estimate for the odds ratio of an association between CYP2C19 LoF variants and previous thrombotic events during clopidogrel therapy indicated an about two-times elevated risk. In 70.1% of patients, CYP2C19 LoF variants led to an evidence-based change of clopidogrel therapy in a clinical setting where a collaboration between clinical pharmacology and vascular specialists had been established.

The dependence of clopidogrel bioactivation and therefore its efficacy on CYP2C19 LoF variants is plausible and has been demonstrated in vitro, and clinical relevance is supported by several prospective randomized studies of high quality [5, 7, 10–16]. On the other hand, non-genetic factors also contribute to outcomes of clopidogrel therapy, the latest generation of drug-eluting stents further reduced the low absolute risk of stent thrombosis under clopidogrel therapy [22], and ticagrelor and prasugrel are alternative antiplatelet drugs without the need of pharmacogenetic testing. Nevertheless, clopidogrel remains the most commonly prescribed P2Y12 inhibitor, and a growing body of evidence including the recent large study by Claassens et al. [5], and at second look also the large study by Pereira et al. [14], support the view that PGx-guided antiplatelet therapy results in the combination of best efficacy and lowest bleeding rates. Therefore, decisions on the implementation of PGx-guided antiplatelet therapy are now mainly driven by cost-effectiveness considerations [15, 23].

In our population, CYP2C19 LoF variants had a prevalence of 32.1%, which is in line with previously reported prevalences for such variants in Caucasian populations [5, 14, 24, 25]. A high prevalence of clinically relevant pharmacogenetic variants supports preemptive PGx testing for all patients receiving clopidogrel, whereas in our referred population, we were able to conduct only 21.4% tests preemptively. However, outside funded systematic studies, preemptive testing requires resources that are to date available only in very few institutions worldwide. In Switzerland, PGx testing for clopidogrel is currently only reimbursed if a clinical pharmacologist confirms its indication. This regulation is based on the recognition that PGx-guided clopidogrel therapy requires more than just a pharmacogenetic test. Indeed, a subanalysis of the GEMINI-ACS-1 multicenter trial reported that mere routine notification of CYP2C19 metabolizer status resulted in switching to another antiplatelet therapy in only 5.9% of patients with current clopidogrel therapy and intermediate or reduced metabolizer genotype [18]. In contrast, in a specialized pharmacogenetic program with integrated clinical decision support at the University of Florida, preemptive testing led to implementation of therapy changes in 70.0% of individuals with actionable genotypes [26]. This closely compares to the implementation rate of 70.1% in our setting. Moreover, a detailed look at the remaining five patients and one additional patient without a recommendation from our study further shows that continued clopidogrel therapies were not just instances of non-compliance. Truly personalized PGx-based pharmacotherapy involves more than automated algorithms as further individual discussions with treating physicians and patients may reveal additional relevant factors. Indications for antiplatelet therapy, non-idiopathic causes for thrombotic events, comorbidities, and comedication have to be weighed against each other for each individual patient. Furthermore, the use of prasugrel is contraindicated in patients with previous intracranial hemorrhage, previous ischemic stroke, transient ischemic attack, or ongoing bleeding, and ticagrelor is also contraindicated in any patients with previous intracranial hemorrhage or ongoing bleeding. For pharmacogenetic expert systems, the implementation of such factors into algorithms is a major challenge. In the study by Claassens et al., 97.6% had DAPT with low-dose aspirin, but only less than 5% concomitant oral anticoagulation, whereas in our population, only 57.1% had aspirin therapy, but 30.4% oral anticoagulants. Our lower proportion of patients with aspirin is explained by the selection of clopidogrel monotherapy as a reason for PGx testing. In our patients with oral anticoagulation, antiplatelet therapy had to be reevaluated with consideration of the latest changing evidence. For example, two meta-analyses published in 2019 and 2020 concluded that anticoagulants should generally not be combined with DAPT [27, 28]. In other patients, the results of the COMPASS trial supported a change to low-dose rivaroxaban plus aspirin as an alternative to clopidogrel [20, 21]. Furthermore, it is also worth noting that in almost 10% of patients our comprehensive clinical pharmacology consultations led to the identification and subsequent change of concomitant drugs that impair the efficacy of clopidogrel through an inhibition of CYP2C19.

The potential benefit of clinical pharmacogenetic services is further supported by the results of our case-control analysis. As much as we would support general preemptive PGx testing for clopidogrel therapy based on recent evidence [5, 14], as long as and where this is not possible, and as long as, e.g., many cardiologists argue that the low absolute risk of thrombotic events has been further diminished with the latest generation of drug-eluting stents, we must develop strategies to focus at least on patients with the highest risk of thrombotic events under clopidogrel. The Clinical Pharmacogenetics Implementation Consortium (CPIC) already recommended testing in moderate or high-risk patients undergoing percutaneous coronary interventions in 2011 [9], and other experts also focus on high-risk populations, at least as long as there are limitations to the availability of PGx testing [8, 15]. According to the study by Claassens et al. comparing PGx-guided therapy vs. ticagrelor or prasugrel as standard, the number needed to test is 125 in order to prevent one ischemic event, 37 to prevent one major bleeding event, and 28 to prevent any of those two outcomes [5]. This is not a high number if one considers that the cost of once in a lifetime PGx testing is low compared to the cost of those complications and that in addition clopidogrel has a lower price compared to ticagrelor or prasugrel. In a high-risk subpopulation of clopidogrel users, the number needed to test is expected to be even lower in order to prevent thrombotic or bleeding events related to CYP2C19 LoF variants. Although the analysis of our case-control analysis is limited by a lack of statistical power, the reported point estimates of an approximately two-times elevated risk of recurrent thrombotic events for CYP2C19 LoF variants are in line with previous studies [5, 7, 10–16]. The risk estimates for other factors are also plausible and can support the identification of subpopulations with the highest absolute risk of thrombotic events under clopidogrel. In a pragmatic approach, we therefore aim to increase efforts to focus on the identification of patients with additional risk factors for the implementation of PGx-guided clopidogrel therapy such as clopidogrel monotherapy without aspirin, diabetes, peripheral arterial disease, stenting of coronary main stem stenosis, or other strong cardiovascular and cerebrovascular risk factors. In the multiplicative model of our logistic regression analysis, a male patient with diabetes and peripheral arterial disease would have an about 20 times elevated baseline risk, and the presence of a CYP2C19 LoF variant would further increase this to a 40 times elevated relative risk of thrombotic events under clopidogrel. Larger future studies should analyze such risk estimates with sufficient statistical power. Other interesting approaches are systematic PGx testing in acute coronary syndromes with the option to later deescalate initial standard therapy with prasugrel to clopidogrel after LoF variants were ruled out [25, 29], or investment into more efficient point-of-care testing on site in cardiac catheter laboratories with rapid availability of genotyping results [5, 14].

In conclusion, in our setting, clinical pharmacology-led implementation of pharmacogenetic services made PGx testing for clopidogrel therapy available to local physicians and their patients, and we currently focus on patients with the highest risk of thrombotic events during clopidogrel therapy. We identified CYP2C19 variants relevant for clopidogrel therapy in more than 30% of tested patients. We achieved a high rate of implementation of PGx-based therapeutic recommendations, but this requires pharmacogenetic as well as cardiovascular expert knowledge and close collaboration with clopidogrel-prescribing specialists, molecular genetics laboratories, pharmacogenetic expert systems, and truly personalized management decisions for each individual patient. Our experience shows that the establishment of such a specialized collaborative expert network must also be anticipated if institutions and health systems plan to establish preemptive PGx testing.
